# An ovarian torsion in a 2-year-old girl: a case report

**DOI:** 10.1186/s13256-020-02518-2

**Published:** 2020-10-18

**Authors:** Mawanane Hewa Aruna Devapriya De Silva, Padmini Kolombage, Sembakutti Kasthuri

**Affiliations:** 1 Department of Paediatrics, Faculty of Medicine, University of Ruhuna, Galle, Sri Lanka; 2 Teaching Hospital, Karapitiya, Galle, Sri Lanka

**Keywords:** Ovarian torsion, Abdominal pain, Oophorectomy, Children

## Abstract

**Background:**

Abdominal pain is one of the most common complaints by patients in the emergency department. Diarrhea, constipation, and urinary tract infection are the commonest etiologies among these patients, but there are surgical emergencies, such as appendicitis and volvulus of the intestine, which are less common. Torsion of the ovary is rarer than all of the above conditions. Ovarian torsion occurs following the twisting of the ovary on its ligamentous attachment, possibly with a cyst, leading to the impediment of blood flow. Prompt diagnosis with a high clinical suspicion is essential to salvage the ovaries and to prevent complications, including death.

**Case presentation:**

Here, we present a case of ovarian torsion in a 2-year-old Sri Lankan girl who presented with nonspecific abdominal symptoms after being symptomatically treated twice by her general practitioners for 3 days. Following biochemical and radiological investigations, she was diagnosed with a twisted necrotic ovarian torsion and underwent laparoscopic right-sided oophorectomy.

**Conclusions:**

Finding the etiology of a child with abdominal pain is challenging, especially because of the limited history, examination findings, the difficulty in carrying out radiological investigations, and the poor specificity of the results compared with adults. This is a case presentation and a brief discussion about the dilemmas and difficulties in the diagnosis and treatment of ovarian torsion in young children.

## Background

Ovarian torsion (OT) is an uncommon gynecological emergency. It is a diagnostic dilemma especially in children, due to non-specificity of the symptoms, overlapping differential diagnosis, and the non-specificity of most of the biochemical and radiological investigations. Hence, a high degree of clinical suspicion and timely investigations are required to confirm the diagnosis. Early diagnosis will salvage the ovary while a delay in diagnosis may lead to ramifications such as loss of ovarian tissue, intra-abdominal sepsis, septicemia, and death.

We report a case of OT in a 2-year-old Sri Lankan girl who presented to our emergency department with nonspecific abdominal pain. Several factors confounded the diagnosis of OT. First, she was seen by her general practitioner twice prior to the presentation at the emergency department. Second, the mistaken belief of the rarity of OT, especially in very young children. Third, our patient initially had clinical and biochemical investigation findings of a urinary tract infection, although it was later proven to be an aseptic pyuria, which is a common presentation in adult patients. And last, our patient’s seemingly benign presentation except for non-specific abdominal pain [1–8].

## Case presentation

A 2-year-old previously healthy Sri Lankan girl presented with lower abdominal pain and low-grade fever. She is from an average Sri Lankan middle class family with no significant family history of ovarian disease and with an average socioeconomic background. She was vaccinated in concordance with the extended immunization schedule of Sri Lanka and there were no significant environmental hazards. She was seen by her general practitioner 3 days prior with postprandial vomiting with no associated diarrhea, constipation, or urinary symptoms. She was otherwise well and was not in pain. She was diagnosed as having acute gastroenteritis. On the third day of the illness, as the symptoms persisted, she was admitted to our hospital for further evaluation.

On examination, she was not in pain but noted to be bent forward while walking. She was comfortable in the seated position and was playful. An examination of her vital signs revealed that they were normal for her age with good hydration and her axillary temperature was 36.2 °C. A cardiovascular system examination revealed a pulse rate of 97 beats/minute, and blood pressure of 96/70 mmHg. Her respiratory rate was 24 breaths/minute and her oxygen saturation 100% on room air without any features suggestive of respiratory distress. The rest of the detailed examination of her cardiovascular and respiratory systems did not reveal any abnormalities. She was rational and conscious with no features suggestive of past or acute neurological deficits. On examination of her abdomen, there was guarding, which was mainly confined to the lower abdomen, equally on both sides. There were no classic clinical features such as severe tenderness on the right inguinal fossa and rebound tenderness to suggest appendicitis. There were no hepatosplenomegaly or ballotable kidneys.

Our patient’s white blood cell count was elevated to 14,700/mm^3^ with 53% lymphocytes and 45% leukocytes. Her hemoglobin and platelet counts were within normal limits. Her C-reactive protein concentration was 42.6 mg/l. Her urine analysis showed 30–40 white blood cells and 2–4 red blood cells. There were trace amounts of protein with significant amounts of ketone bodies. Therefore, on the day of admission, a tentative diagnosis of urinary tract infection was made and she was started on intravenous gentamicin 5 mg/kg/day. A urine culture did not grow any organisms. There was no bacterial growth in her blood culture. Renal function, liver profiles, serum electrolytes, and other basic biochemical investigation results were within normal limits.

**Fig. 1:**
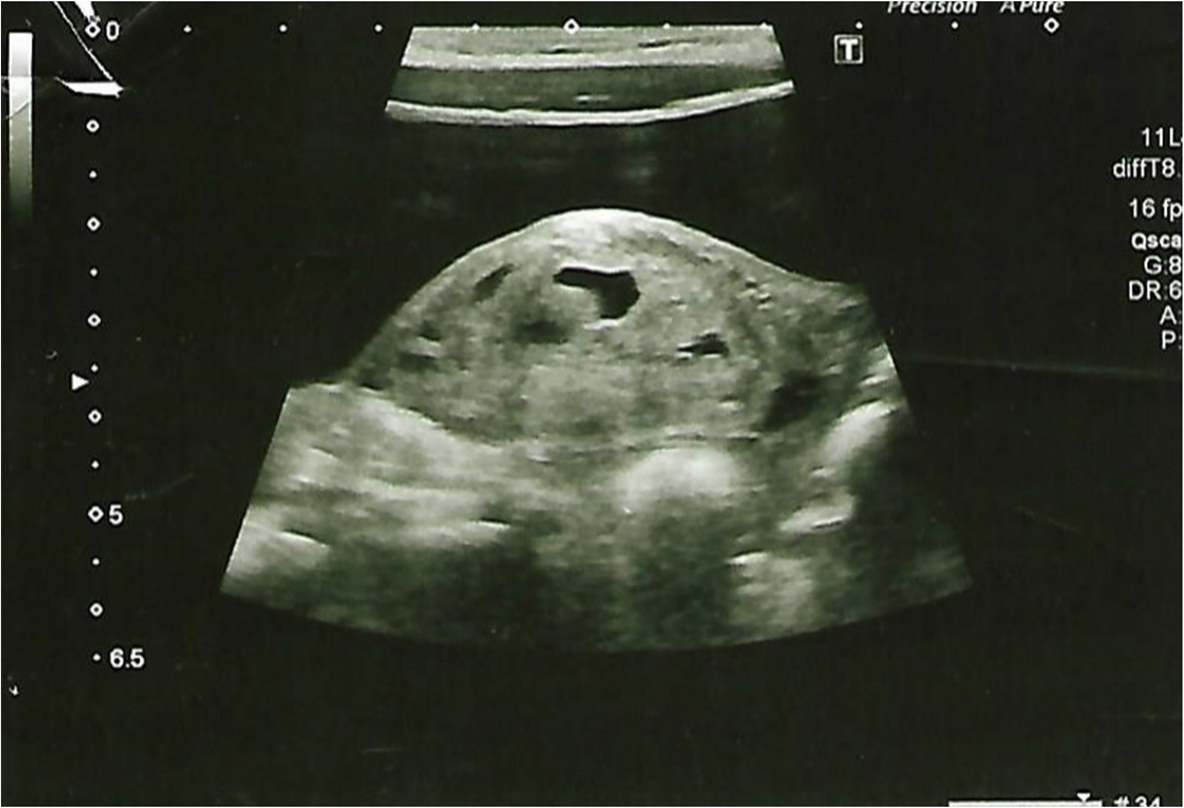
Hyperechoic mass on the pouch of Douglas on right side measuring 5 cm × 2.5 cm × 3 cm suggestive of ovarian cyst

**Fig. 2:**
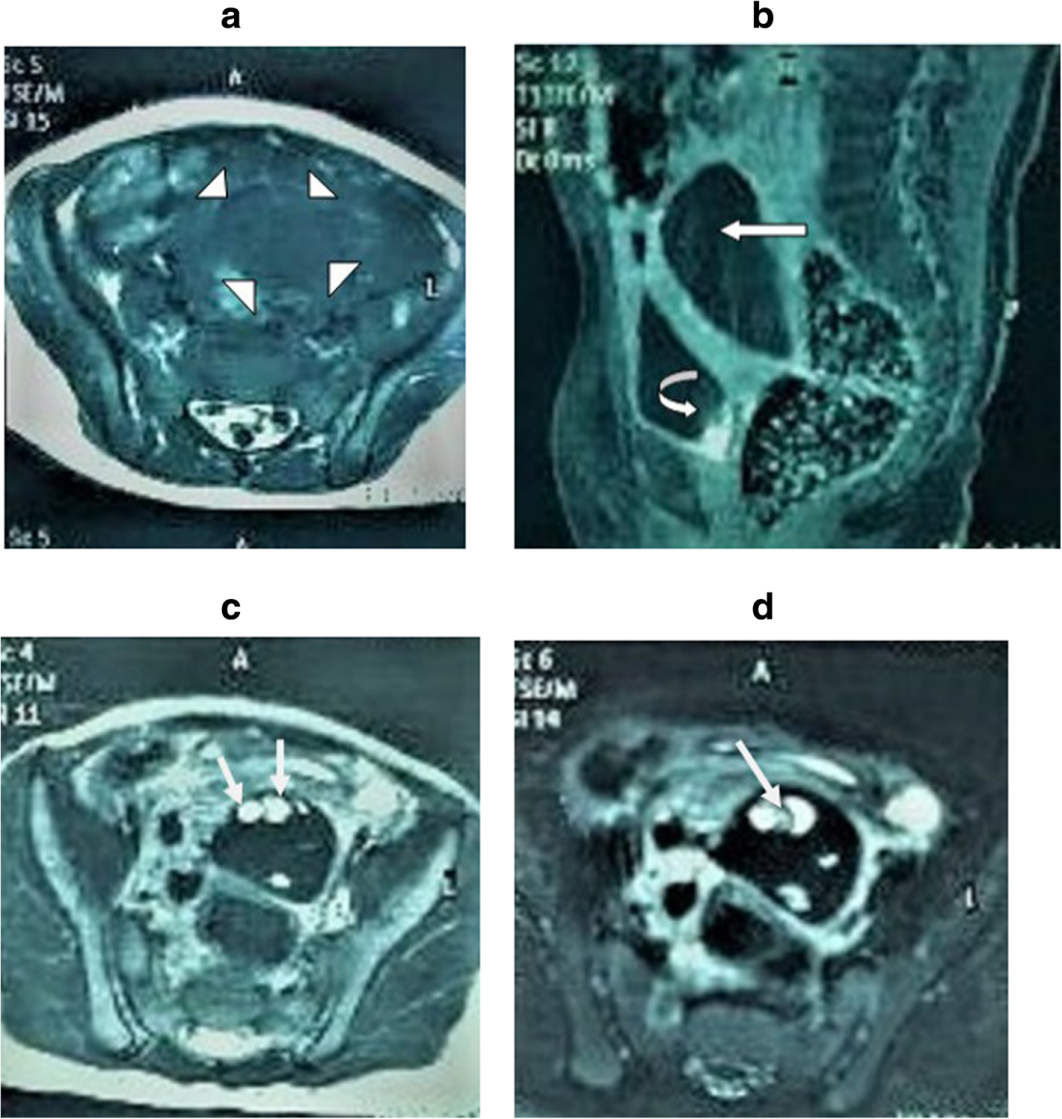
Torsion of the right ovary of a 2-year-old girl with a history of abdominal pain and vomiting. **a** Axial T1-weighted MR image shows enlarged right ovary (arrowheads). **b** Sagittal contrast-enhanced fat-suppressed T1-weighted MR image shows no contrast enhancement in the right ovary (arrow). Contrast is seen within the urinary bladder (curved arrow). **c** Axial T2-weighted MR image shows multiple cystic areas within the enlarged right ovary favoring enlarged follicles (arrows). **d** Axial T2-weighted fat-suppressed MR image shows hemorrhages of varying stages (arrow). MR magnetic resonance

An abdominopelvic ultrasound scan revealed a mixed but predominantly hyperechoic mass on the pouch of Douglas on her right side measuring 5 cm × 2.5 cm × 3 cm suggestive of ovarian cyst (Fig. 1). Her left ovary was normal. There was no evidence of appendicitis or pyelonephritis. A magnetic resonance imaging scan of her abdomen revealed an enlarged right ovary with hemorrhages at various stages with multiple enlarged follicles within it, which was suggestive of OT (Fig. 2a–d). It did not show any enhancement with intravenous gadolinium.

**Fig. 3:**
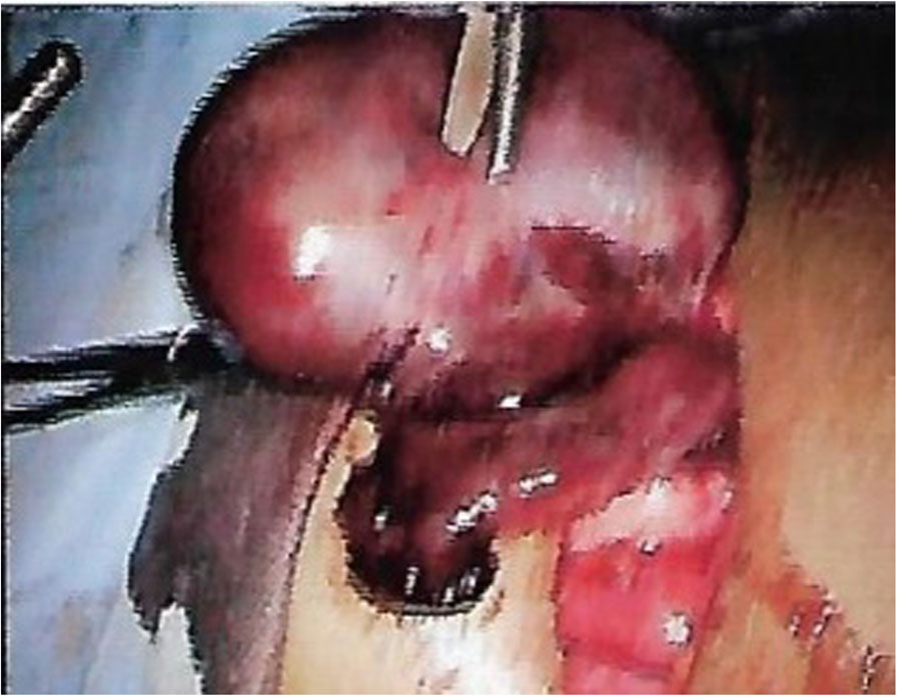
Twisted gangrenous right ovary

Exploratory laparoscopy revealed a twisted gangrenous right ovary (Fig. 3) following which an oophorectomy was performed as no viable ovarian tissue was noticed. Our patient recovered with no complications. Histology of the specimen revealed features suggestive of a benign ovarian torsion undergoing torsion with venous infarction.

After 36 hours, gentamicin was stopped and she was started on intravenous cefotaxime 50 mg/kg/dose every 6 hours and intravenous metronidazole 7.5 mg/kg/dose every 8 hours for 5 days.

The follow-up at 12 months revealed no complications or any other major illnesses. Her growth and development were also in concordance with the mean for her age and sex.

## Discussion

Adult and adolescent patients with OT present with typical clinical features. The younger the patient, the more non-specific the features become. Diagnosis becomes more and more challenging for the treating clinician, especially in a resource-limited setting. Our patient presented with clinical and initial biochemical investigation findings of a urinary tract infection with a negative urine culture proving it to be an aseptic pyuria. Although aseptic pyuria has been reported in adolescent cases, this case reiterates the importance of investigating younger patients with aseptic pyuria further for the possibility of OT, especially when the patient’s symptoms persist or worsen [1–8].

OT is a rare but an important and mandatory differential diagnosis in a female patient who presents with abdominal pain. It is reported as the fifth most common gynecological emergency. It has a prevalence of 2.7% and an incidence of 4.7 per 100,000 patients in women less than 20 years old [1–4]. The highest prevalence is in women of reproductive age [5, 6]. Pediatric and adolescent cases of OT account for about 15% of the total cases. About 52% of pediatric and adolescent cases of OT occur between 9 and 14 years of age with a mean age of about 11 years. Cases of OT in infants is rare with only 16% of cases occurring in girls under 1 year old [7, 8]. The majority of OT occurs on the right side due to less space for twisting of the left ovarian tissue because of the sigmoid colon and relatively long right ovarian ligament enabling it to twist [9, 10].

Diagnosis of OT is clinical, but the confirmation of the diagnosis is made at the exploratory surgical procedure. It is challenging to a primary physician or a pediatrician not only because of its rarity, but also because of the non- specificity of the clinical and investigation findings. Children with OT usually present with nonspecific right-sided lower abdominal pain, which might be variable in nature with a constant or intermittent, non-radiating or radiating, mild or intense pain, and with a variable duration from a few hours to days. Other symptoms like vomiting and nausea may mimic the common presentation of acute gastroenteritis, constipation, or urinary tract infection. Surgical conditions like appendicitis and intussusception also overlap with the clinical picture.

Examination findings such as lower abdominal tenderness and fever will also be nonspecific. A Doppler ultrasound scan would be the most important investigation [11]. In adults, a transvaginal scan is performed and in children, it is performed through the abdominal wall, which has less sensitivity. Typically, a Doppler scan reveals diminished or absent vascular flow [11]. However, presence of vascular flow does not exclude OT. The study itself is difficult the younger the patient and the specificity of the findings will be less, It makes for a diagnostic dilemma, especially in very young patients. Magnetic resonance imaging and computed tomography are more sensitive options in diagnosing and ruling out the other differentials, but it is not feasible in a resource-poor setup. Full blood count, inflammatory markers, renal function tests, liver function tests, and urinary analysis are done in most of the suspected cases of OT, mainly to exclude other differential diagnoses [12, 13]. Aseptic pyuria is recognized in some patients [9].

In the case we discussed, our patient presented with clinical and initial biochemical investigation findings of a urinary tract infection, but later it proved to be aseptic pyuria as the urine culture was negative. Therefore, it is recommended to investigate for the possibility of OT in the case of aseptic pyuria, especially when patient symptoms persist or worsen. Although aseptic pyuria has been reported in adolescent cases, this is the youngest child reported with similar symptoms [14].

Rare as it may be, OT should be considered in order to salvage ovarian function in every female child who presents with abdominal pain.

## Conclusions

High clinical suspicion with prompt timely investigations and emergent surgical intervention is the key to salvage ovarian function with minimum morbidity. In a resource-limited setting, it is a challenge to the clinician to confirm the diagnosis within a very limited time frame. Our patient had to undergo oophorectomy, but she did not have other complications. The younger the age, the more difficult the diagnosis, but with any features suggestive of significant unexplained abdominal pain in a girl, OT should be always excluded with certainty within the earliest possible time frame.

## Data Availability

Not applicable.

## Abbreviation

OT: Ovarian torsion
